# Supplementary material for: First-in-Class Humanized Antibody against Alternatively Spliced Tissue Factor Augments Anti-Metastatic Efficacy of Chemotherapy in a Preclinical Model of Pancreatic Ductal Adenocarcinoma
Source: Int J Mol Sci. 2024 Feb 23;25(5):2580. doi: 10.3390/ijms25052580 (PMC10932375; doi:10.3390/ijms25052580)
Supplement: Supplementary file 1 [file ijms-25-02580-s001.zip › ijms-2829842-supplementary.pdf]

Supplementary Figures

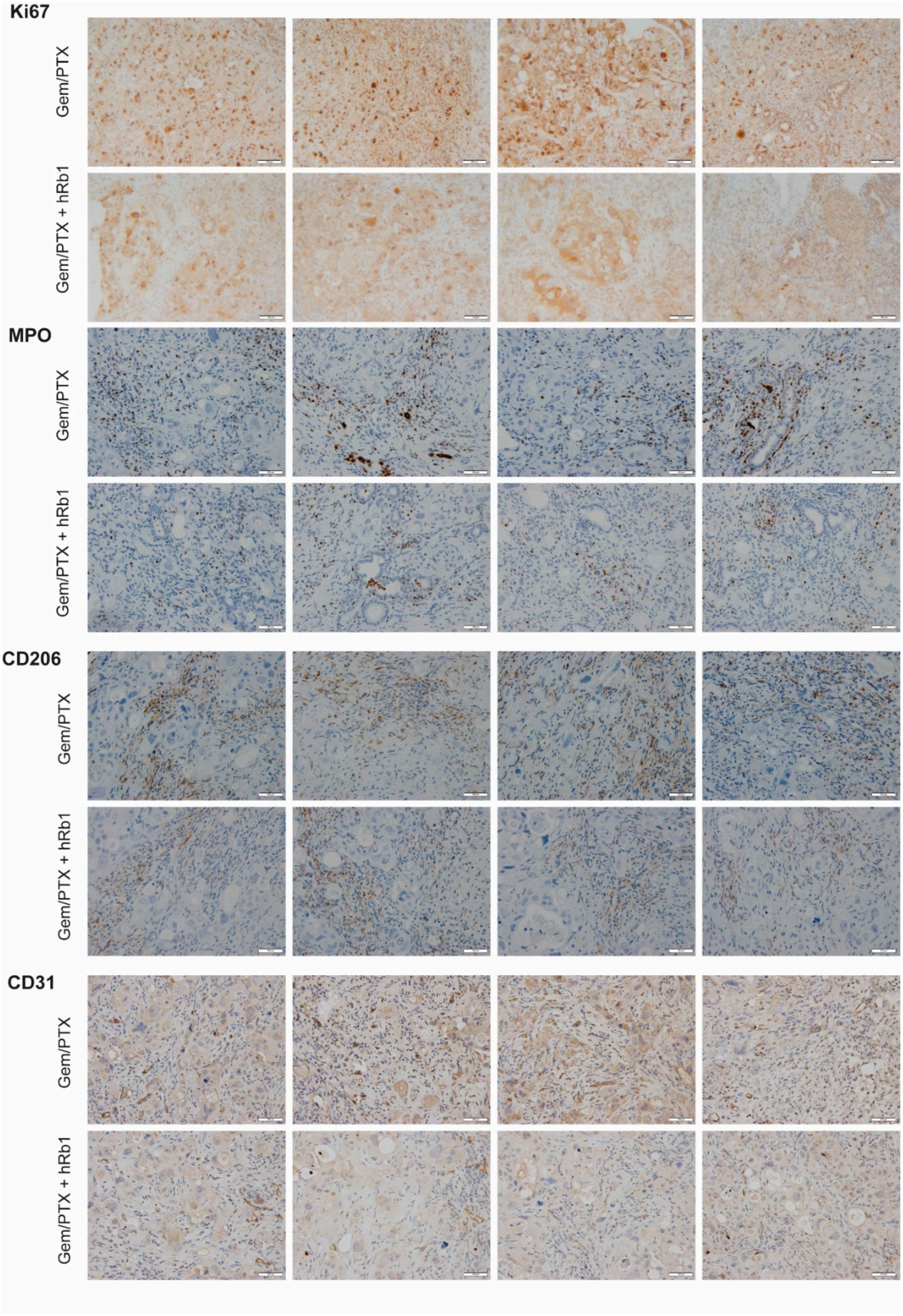

Supplementary Figure S1. Representative images, IHC of primary tumor tissue stained for the indicated markers (Ki67, MPO, CD206, CD31); original magnification 20x. 100 μm scale bar shown in bottom right of each micrograph.

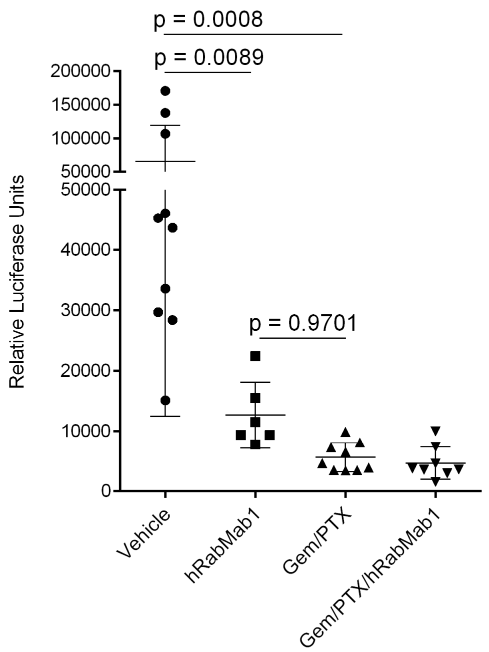

Supplementary Figure S2. Relative luciferase units, metastatic spread to the liver assessed by quantitative luciferase imaging in experimental cohorts as indicated; 1-way ANOVA with Tukey's multiple comparison test was used to assess significance. Liver tissue was harvested and imaged immediately following the removal of primary tumors.

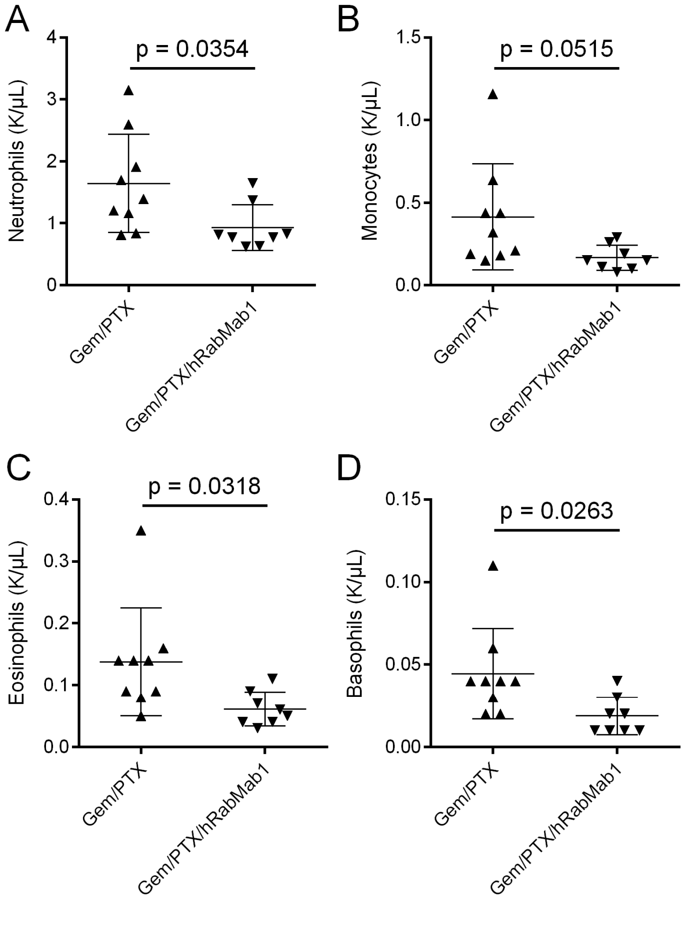

Supplementary Figure S3. White blood cell counts in Gem/PTX and Gem/PTX/hRabMab1 cohorts (1 dot = 1 mouse): A, neutrophils; B, monocytes; C, eosinophils; D, basophils. Whole blood was collected via venipuncture at the time of sacrifice in EDTA-coated tubes; two-tailed t test was used to assess significance.
